# Alarmin Levels and Gastroesophageal Reflux Disease in Children: Significant Elevation of Thymic Stromal Lymphopoietin

**DOI:** 10.3390/pediatric17050093

**Published:** 2025-09-15

**Authors:** Ola Sobieska-Poszwa, Szymon Suwała, Aneta Mańkowska-Cyl, Aneta Krogulska

**Affiliations:** 1 Department of Paediatrics, Allergology and Gastroenterology, Ludwik Rydygier Collegium Medicum Bydgoszcz, Nicolaus Copernicus University Torun, 85-094 Bydgoszcz, Poland; aneta.krogulska@cm.umk.pl; 2 Department of Endocrinology and Diabetology, Ludwik Rydygier Collegium Medicum Bydgoszcz, Nicolaus Copernicus University Torun, 85-094 Bydgoszcz, Poland; szymon.suwala@abs.umk.pl; 3 Department of Laboratory Diagnostic, Ludwik Rydygier Collegium Medicum Bydgoszcz, Nicolaus Copernicus University Torun, 85-094 Bydgoszcz, Poland; aneta.mankowska@cm.umk.pl

**Keywords:** gastroesophageal reflux disease, TSLP, IL-25, IL-33, periostin

## Abstract

**Background/Objectives**: In children, gastroesophageal reflux disease (GERD) may lead to epithelial barrier dysfunction and the release of thymic stromal lymphopoietin (TSLP), interleukin-25 (IL-25), interleukin-33 (IL-33) and periostin, known as alarmins. These cytokines are associated with type 2 inflammation and may contribute to respiratory and allergic conditions. The main purpose of this study is to evaluate serum concentrations of TSLP, IL-25, IL-33, and periostin in children with and without GERD and to assess their relationships with bronchial hyperresponsiveness (BHR) and sensitization to inhaled allergens. **Methods**: The study included 93 children aged 7–17 years. GERD was diagnosed based on 24-h esophageal pH impedance monitoring. Serum levels of TSLP, IL-25, IL-33, and periostin were measured using enzyme-linked immunosorbent assay (ELISA). It should be noted that the assay used does not distinguish between TSLP isoforms, which represents a limitation of the study. BHR was assessed via a methacholine challenge test, and allergen sensitization was determined using skin prick tests and allergen-specific immunoglobulin E (asIgE). **Results**: Serum TSLP levels were significantly higher in children with GERD compared to those without, whereas IL-25, IL-33 and periostin did not differ notably between groups. Periostin was associated with the degree of sensitization to inhalant allergens, but no significant links were found between cytokine levels and bronchial hyperresponsiveness. **Conclusions**: Significantly higher TSLP levels were noted in children with GERD than in those without. Hence, TSLP may have a potential role as a biomarker of epithelial immune activation in pediatric GERD. In addition, periostin was associated with sensitization to inhalant allergens, although it did not differentiate between children with and without GERD.

## 1. Introduction

Mechanical injury, infection, inflammatory cytokines and proteases such as trypsin and papain stimulate the release of various cytokines of epithelial origin, such as thymic stromal lymphopoietin (TSLP), interleukin-25 (IL-25), interleukin-33 (IL-33) and periostin. As these cytokines are produced as the first line of defense against infections and other stimulators of the respiratory epithelium, they are known as alarmins. Their presence is associated with increased allergic inflammation, and appears to drive interactions between the innate and acquired immune systems [1,2,3]. They are also known to be involved in the pathogenesis of a range of allergic diseases, including asthma, atopic dermatitis and food allergy by activating Th2-dependent immune responses [3]. A better understanding of the influence of alarmins on the course of these conditions would offer hope for the introduction of new treatments using biological drugs, and could represent a breakthrough in determining prognosis; however, this knowledge remains unclear [3,4]. We therefore focused on the epithelial alarmins TSLP, IL-25 and IL-33, which are important initiators and amplifiers of type 2 inflammation, and on periostin, a biomarker of Th2-driven remodeling. These molecules were specifically selected because of their established relevance in pediatric airway disease. In children with asthma, TSLP, IL-25 and IL-33 are recognized as key epithelial mediators that define the T2-high endotype and have been highlighted as potential therapeutic targets and biomarkers of disease activity [5]. Periostin, in turn, has been reported to be elevated in the serum of children with asthma, supporting its utility for diagnosis and patient stratification [6]. In this study, we measured total TSLP level without distinguishing its isoforms, which should be considered a methodological limitation.

Many conditions are underpinned by disorders in homeostasis. In such cases, homeostasis is maintained by the state of the epithelium: disruption of the epithelial barrier has been shown to be a key element in the pathogenesis of many diseases, including allergic diseases. Such abnormalities in the epithelial barrier are known to result from esophageal diseases such as gastroesophageal reflux disease (GERD) or eosinophilic esophagitis (EoE).

Furthermore, numerous studies indicate that certain gastrointestinal diseases may be associated with allergic diseases, as exemplified by the co-occurrence of asthma with GERD [7]. It has been shown that inflammatory changes within the respiratory tract in asthma can be induced or exacerbated by gastroesophageal reflux (GER). Three etiologies have been proposed to account for the induction of respiratory inflammation by GERD: the direct (microaspiration) theory, the indirect (reflex) theory and the neurogenic inflammation theory [8,9,10].

GERD is a significant gastrointestinal disorder both in adults and in children. The disease is characterized by frequent recurrences and complications such as impaired physical development, recurrent respiratory disorders or changes in the epithelial lining of the distal part of the esophagus, in the form of Barrett’s esophagus. Typically, the disease requires long-term treatment, whose effectiveness is indicated by the resolution of symptoms. Therefore, there is a pressing need for improved diagnostic tools and more effective therapeutic approaches for managing this condition.

EoE is less prevalent than GERD: EoE is estimated to affect 4.29 per 10,000 children [11], compared to 480 per 10,000 in GERD [12]. Typical symptoms of GERD, such as heartburn, epigastric pain, chest pain, dysphagia, nocturnal pain, regurgitation and acid reflux, can often be accompanied by atypical symptoms, some of which are associated with the respiratory system, such as nocturnal coughing, wheezing, recurrent pneumonia, sore throat, hoarseness and chronic sinusitis [13,14].

Despite growing interest in the role of alarmins, such as TSLP, IL-25, IL-33 and periostin, in allergic diseases, most existing data have been acquired from studies of adults or eosinophilic esophagitis, and little is known of their activity in children with GERD. Clarifying these mechanisms in the pediatric population may support the development of early diagnostic markers or targeted treatments. A key role in initiating Th2-type immune responses is played by TSLP, an epithelial-cell–derived cytokine. Two isoforms of TSLP have been described in the human body: a long form (lfTSLP), consisting of 159 amino acids, and a short form (sfTSLP), consisting of 63 amino acids. The long form is generally induced by proinflammatory stimuli and is associated with chronic inflammatory conditions such as asthma and atopic dermatitis. In contrast, the short form is constitutively expressed and may be involved in maintaining epithelial barrier homeostasis. TSLP is primarily produced in epithelial cells of the skin, lungs, and gastrointestinal tract [15,16,17]. Despite their clinical relevance, TSLP isoforms are often not distinguished in immunoassays; this can limit their potential for interpretation, including in the present study.

In children, GERD is most commonly associated with functional immaturity of the lower esophageal sphincter, delayed gastric emptying, congenital anomalies (such as esophageal atresia with surgical repair), neurological impairment, and the presence of hiatal hernia. Obesity may also play a role in older children and adolescents. In contrast to adults, factors such as pregnancy and smoking are less relevant in pediatrics.

The cytokines IL-25, IL-33, and periostin are involved in type 2 immune responses and epithelial barrier function. IL-25, also known as IL-17E, is produced primarily by epithelial cells and enhances Th2-type immune responses, promoting eosinophilia, IgE production, and mucus secretion. IL-33, a member of the IL-1 cytokine family, is released upon cell damage or stress and acts as an alarmin, initiating inflammation and contributing to allergic diseases and asthma. Periostin is an extracellular matrix protein secreted by epithelial and fibroblast cells under the influence of IL-4 and IL-13; it enhances eosinophilic inflammation and tissue remodeling in allergic conditions. These mediators play a central role in allergic sensitization, asthma, and chronic inflammatory diseases.

As the epithelium plays an important role in the pathogenesis of both allergic and esophageal diseases, and alarmins are known to influence the development of allergies, as well as the repeatedly documented link between GERD and asthma or food allergy, the main aim of the study was to determine the levels of selected alarmins, viz. TSLP, IL-25, IL-33 and periostin, in children with GERD. An additional aim was to evaluate alarmin levels in relation to the presence of bronchial hyperresponsiveness and the degree of sensitization to inhalant allergens. It would also be worthwhile to clarify whether acidic or non-acidic reflux contents damage the esophageal epithelium, leading to the release of alarmin cytokines and periostin. While studies have evaluated the importance of alarmins in EoE, no such studies have concerned GERD.

## 2. Materials and Methods

### 2.1. Patients

**Figure 1:**
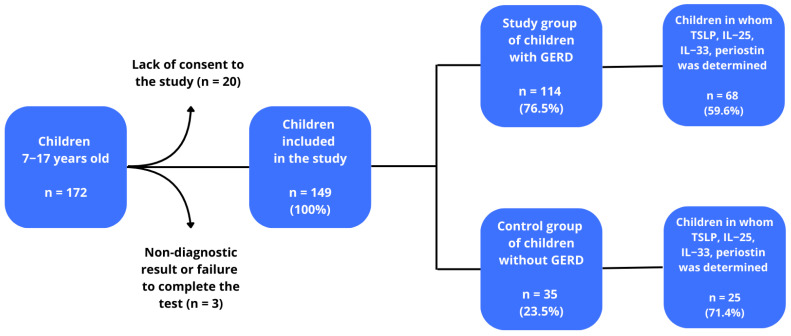
Patient eligibility scheme for the study.

The study included children with suspected GERD. All had been hospitalized at the Department of Paediatrics, Allergology and Gastroenterology and had been under the care of the Gastrological Outpatient Clinic between 1 October 2017 and 31 March 2020. Initially, 172 children were included in the study. Twenty-three patients were excluded: 20 due to lack of consent and three due to non-diagnostic test results or failure to complete the study, lack of blood samples or technical errors. After taking into account the above aspects, 149 children were enrolled to the next stage of selection in the study. These children were divided into a group of 114 children diagnosed with GERD (76.51%), and a group of 35 without GERD (23.49%). TSLP, IL-25, IL-33 and periostin levels were determined in 68 children with GERD (59.6%) and in 25 children without GERD (71.4%), and this group of patients constituted the final study group. Finally 68 (73.1%) children were included as a study group, and 25 (26.9%) as a control group (the small number of control study participants may be a potential limitation of the study, but this number meets the criterion of the minimum sample size *n* ≥ 23). Among the GERD group, six children demonstrated borderline bronchial hyperresponsiveness, six the mild form, and two the significant form, while no hyperreactivity was observed in the remaining 40. Among those without GERD, one child demonstrated mild bronchial hyperresponsiveness, and two the borderline form, while 13 did not present any disease. The diagnosis of GERD was based on clinical interview and confirmed using 24-h esophageal pH impedance monitoring [18,19]. Data on current medication use, including treatments for allergy and asthma, were collected through a detailed medical interview. The GERD group included both allergic and non-allergic individuals, with a number of members being obese. A flow chart of the study is shown in Figure 1.

The inclusion and exclusion criteria are presented in Table 1.

Patient characteristics, including socio-demographic data, are presented in Table 2; no statistically significant differences were observed between the study and control groups (*p* < 0.05).

### 2.2. Survey Analysis

Patients were qualified for the study on the basis of a modified version of the Gastroesophageal Reflux Disease Questionnaire-Q (GerdQ): a questionnaire of GERD-related symptoms [20]. Each patient also completed a survey based on the validated International Study of Asthma and Allergies in Childhood (ISAAC) questionnaire [7], again with our own modification. Both questionnaires were completed by the parents of the children surveyed, and in the case of older children, the parents completed the questionnaire together with the patients.

### 2.3. Intra-Esophageal pH Impedance Measurements

Gastroesophageal reflux was assessed by 24-h pH impedance monitoring; this was performed in a hospital setting using an instrument and polyvinyl probes from Sandhill Scientific Inc (Highlands Ranch, CO, USA). A diagnosis of GERD was confirmed using 24-h esophageal pH impedance monitoring based on standard cut-off values: GERD was indicated by an esophageal pH below 4 for more than 6% of the time in older children, and more than 12% in younger children. Additionally, a total number of reflux episodes exceeding 75 was also considered indicative of pathological reflux. It is worth noting that although a pH threshold of 4.0 is most commonly applied, some experts propose using 5.0, or even a broader range between 3.0 and 6.0, to better distinguish physiologic from pathologic reflux. Moreover, pH impedance monitoring increases diagnostic sensitivity by detecting weakly acidic and non-acid reflux episodes and can provide additional indices such as symptom index (SI), symptom sensitivity index (SSI), and symptom association probability (SAP), which help correlate symptoms with reflux events [18,21].

### 2.4. Evaluation of Serum Levels of IL-33, IL-25, TSLP and Periostin

Serum levels of IL-33, IL-25, TSLP and periostin were determined by collecting approximately 4 mL of venous blood. The sample was collected approximately 12 h after the last meal, with the blood being drawn into sterile tubes without anticoagulant to allow clotting. The samples were left at room temperature for 30 min to achieve complete coagulation. The tubes were then centrifuged at 3000× *g* rpm for 15 min to obtain the serum. The serum was transferred to Eppendorf-type tubes and stored at a temperature below −70 °C for a maximum of 6 months. The test material was thawed immediately before the assay. Serum alarmin concentrations were determined by “sandwich” immunoenzymatic ELISA using Enzyme-Linked Immunosorbent Assay Kit reagents from Cloud-Clone Corp., Houston, TX, USA. Assay procedures were performed according to the manufacturer’s instructions. The manufacturer’s reported coefficient of variation was less than 10% (intra-assay) and less than 12% (inter-assay) for IL-33, IL-25, TSLP and periostin. The limit of detection (LoD) of the assay was 0.115 pg/mL for IL-33, 0.05 pg/mL for IL-25, 0.58 pg/mL for TSLP and 0.056 pg/mL for periostin, respectively.

### 2.5. Assessment of Bronchial Hyperresponsiveness

Bronchial hyperresponsiveness (BHR) testing was carried out using a methacholine challenge with the Lungtest 1000 spirometer and ISPA software (MES, Kraków, Poland). The methacholine challenge was performed in accordance with European Respiratory Society (ERS) recommendations [19]. Patients inhaled increasing concentrations of methacholine chloride dissolved in physiological saline: 0.0625 mg/mL, 0.25 mg/mL, 1.0 mg/mL, 4.0 mg/mL, and 16 mg/mL. Each dose was administered via tidal breathing over 2 min, followed by spirometric measurement of forced expiratory volume in 1 s (FEV1). The test continued until a 20% decrease in FEV1 was achieved or the highest concentration was administered without any increase being observed. The degree of BHR was classified as follows: severe BHR when PD20 < 0.1 mg, moderate BHR 0.1–1.0 mg, mild BHR 1.0–4.0 mg, borderline BHR 4.0–8.0 mg, and no BHR when PD20 > 8.0 mg; PD20 is the provocative dose causing a 20% fall in FEV1.

### 2.6. Measurement of Exhaled Nitric Oxide Levels (FeNO)

The concentration of nitric oxide exhaled with air from the bronchi (FeNO) was determined using a Hypair FeNO device (Medisoft, Soriness, Belgium). At least two FeNO measurements were performed in each patient, and the mean value was taken as the final result. The examination was conducted in accordance with the recommendations of the American Thoracic Society (ATS) [22,23].

### 2.7. Assessment of Sensitization to Airborne Allergens

Sensitization was assessed by skin prick test (SPT) and allergen-specific IgE (asIgE) level in the blood. Sensitization was defined as the identification of at least one positive skin test or the presence of at least one asIgE at a concentration ≥0.35 kU/L. A single observed sensitization indicates one positive SPT and/or the presence of at least one asIgE at a concentration ≥0.35 kU/L. Similarly, two observed sensitizations indicate two positive SPTs and/or the presence of at least two asIgEs at a concentration ≥0.35 kU/L.

The skin prick test was performed using the following allergens: grass pollen, rye, alder, hazel, birch, mugwort, plantain, Dermatophagoides pteronyssinus, Dermatophagoides farinae, dog epidermis, cat epidermis and mold (Alternaria alternata, Aspergillus fumigatus, Cladosporium herabarum). The test used standardized reagents from Allergopharma—Nexter (Reinbek, Germany), according to European Academy of Allergy and Clinical Immunology (EAACI) recommendations [24].

The concentration of allergen-specific IgE (asIgE) in blood serum was determined by the Polycheck method (Biocheck GmbH, Munster, Germany) for allergens: birch pollen, alder, hazel, timothy, rye, mugwort, plantain, *D. pteronyssinus*, *D. farinae*, dog epidermis, cat epidermis, Aspergillus fumigatus, Cladosporium herabarum, Pencillium notatum and Alternaria alternata. Sensitization was indicated by the presence of asIgE against a given allergen at a titer ≥ 0.35 kU/L.

### 2.8. Statistical Analysis

Quantitative variables are presented as mean ± standard deviation (SD), and qualitative variables as proportions. The quantitative variables were checked for a normal distribution using the Kolmogorov–Smirnov test: pairs with a normal distribution were compared using the Student’s *t*-test, and those with a non-normal distribution using the Mann–Whitney U-test. The three groups were compared using an analysis of variance (ANOVA) with either the least significant difference test (NIR) or a Kruskal–Wallis post hoc test, according to the distribution. Qualitative variables were compared using a chi-square test or Fisher’s exact test as appropriate. A value of *p* < 0.05 was assumed as statistically significant. All calculations were carried out using STATISTICA 13.0, Polish version.

### 2.9. Institutional Review Board Statement

The study was approved by the Bioethics Committee of the Ludwik Rydygier Collegium Medicum in Bydgoszcz, Nicolaus Copernicus University in Toruń, Poland (Bioethics Committee Headquarters: ul. M. Skłodowskiej-Curie 9, 85-094 Bydgoszcz, Poland). Ethical approval was granted on 26 September 2017 (No. KB 643/2017) and remained valid throughout the study duration until its conclusion in March 2020.

## 3. Results

TSLP, IL-25, IL-33 and periostin levels were determined in all children: 68 in the study group (73.1%) and 25 in the control group (26.9%). TSLP concentrations ranged between 0.875 and 74.080 pg/mL: mean value 19.32 ± 11.62 pg/mL. It was found that the mean TSLP concentration was significantly higher in children with GERD (21.5 ± 11.7 pg/mL) compared to those without GERD (13.3 ± 9.0 pg/mL; *p* = 0.002). IL-25 concentrations ranged from 1.079 pg/mL to 72.456 pg/mL, with a mean of 14.27 ± 24.58 pg/mL. No significant difference was noted in mean IL-25 concentrations between the children with GERD (13.71 ± 13.48) and those without GERD (8.07 ± 7.72; *p* = 0.151). IL-33 concentrations ranged between 0.124 pg/mL and 20.273 pg/mL, mean 6.68 ± 4.71 pg/mL. Mean IL-33 concentrations were similar in both children with GERD (6.9 ± 4.9) and those without GERD (6.2 ± 4.2; *p* = 0.767). Periostin concentrations varied between 0.740 pg/mL and 18.089 pg/mL, with a mean of 5.79 ± 2.41 pg/mL. Mean periostin levels were similar in both groups of children, with GERD (5.9 ± 2.7 pg/mL) and without GERD (5.5 ± 1.4 pg/mL; *p* = 0.994).

Regarding the influence of acidity, it was found that all alarmin mean concentrations were similar in children with acid reflux (*n* = 32) and non-acid reflux (*n* = 36): IL-25 11.9 ± 13.7 pg/mL in acid reflux vs. 15.2 ± 14.9 pq/mL in non-acid reflux (*p* = 0.362); TSLP—20.0 ± 11.5 pg/mL vs. 21.6 ± 12.9 (*p* = 0.603); IL-33—6.8 ± 4.7 pg/mL vs. 6.9 ± 5.1 pg/mL (*p* = 0.953); periostin—5.4 ± 2.0 pg/mL vs. 6.3 ± 3.2 pg/mL (*p* = 0.168). The observed effect sizes were small (rank-biserial correlation <0.18 for all comparisons); however, with the available sample, the post hoc power to detect these small effects was low (<0.25).

Bronchial hyperresponsiveness (BHR) affected 31 (33.3%) children, including 22 (32.4%) with GERD and seven (28.0%) without GERD. Borderline hyperreactivity was noted in six (8.8%) children in the study group, but in no children in the control group. Mild hyperreactivity was demonstrated in seven (10.3%) children with GERD and three (12.0%) without GERD, while moderate hyperreactivity was noted in 16 (23.53%) children with GERD compared to three without GERD (12.0%) (*p* = 0.347). No severe hyperreactivity was found in any of the children. No significant relationship was observed between TSLP, IL-25, IL-33, periostin and bronchial hyperresponsiveness in either the study or control group (Table 3).

The study also analyzed the mean concentrations of TSLP, IL-25, IL-33 and periostin with regard to the prevalence of sensitization to inhalant allergens. A statistically significant difference was found only in periostin values, which were significantly higher in sensitized children (evaluated only by asIgE) with GERD (7.61 ± 4.11 pg/mL) compared to non- sensitized children with GERD (5.34 ± 1.72 pg/mL) (*p* = 0.002). No statistically significant differences were found with regard to TSLP, IL-25 or IL-33 (Table 4 and Table 5).

The study also examined the relationship between the concentrations of TSLP, IL-25, IL-33 and periostin and the number of sensitizations to inhalant allergens (assessed by asIgE) present in children. The mean periostin concentration was 6.8 ± 1.3 pg/mL in children with sensitization to one allergen, 7.6 ± 4.1 pg/mL in children with sensitization to two to five allergens and 4.5 ± 1.5 pg/mL in children with sensitization to more than five allergens (*p* = 0.003) (Table 6).

Concentrations of TSLP, IL-25, IL-33 and periostin were also analyzed according to the amount of sensitization present, with regard to GERD status. Among children with sensitization, periostin concentrations were found to be significantly higher with those with co-occurring GERD, compared to those without GERD. Values among children with GERD were 5.34 ± 1.72 pg/mL in those without sensitization, 7.22 ± 1.38 pg/mL in those with sensitization to one allergen, 8.43 ± 4.74 pg/mL with sensitization to two to five allergens, and 3.79 ± 1.66 pg/mL in children with sensitization to more than five allergens (*p* = 0.002).

## 4. Discussion

Our findings indicate significant differences between children with GERD and those without GERD with regard to TSLP levels, but no such differences exist for IL-25, IL-33 or periostin. In addition, an association was found between IL-25 level and type of reflux, and a correlation between periostin levels and sensitization in children with GERD.

These observations can be accounted for by the pathomechanism of the disease. In children, as in adults, the main pathogenesis of GERD is abnormal transient relaxation of the lower esophageal sphincter. However, it can also be influenced by the anatomy and integrity of the reflux barrier and some aspects of esophageal peristalsis and clearance [25]. In addition, the esophageal mucosal defense may be impaired in cases of esophagitis, or by damage caused by gastric reflux. A defect in the esophageal mucosal defense can lead to esophageal motility disorders and can be superimposed on reflux esophagitis [26,27].

Like many parts of the body, the esophageal epithelium is subject to homeostatic disorders driven by a number of factors and agents, such as acids and inflammatory cytokines; it is also affected by growth factors which activate various signaling pathways regulating esophageal epithelial cell function. Indeed, esophageal fibroblasts are known to produce cytokines in response to a variety of factors and agents [28].

The release of tissue-associated cytokines such as TSLP, IL-33 and IL-25 from epithelial and stromal cells plays a key role in the initiation and maintenance of tissue immunity. In addition, these molecules also influence both canonical type 2 responses and homeostasis [27].

Both EoE and GERD are characterized by abnormalities of the esophageal epithelial barrier. However, while previous studies have evaluated the importance of alarm cytokines in EoE, no such research has addressed their role in GERD. Our findings indicate that while children with GERD tend to have higher TSLP values than those without GERD, no relationship was observed between TSLP level and reflux type. This may suggest that TSLP release, and thus the development of esophageal inflammation, is induced by damage to the esophageal epithelium caused by retreating gastric contents, irrespective of their acidity.

### 4.1. TSLP

For over two decades, TSLP has been investigated as a mediator of inflammatory processes in the respiratory tract, gastrointestinal system, and skin. Its expression is elevated in the airways of patients with asthma, correlating with disease severity and impaired lung function [29,30]. TSLP production can be induced in the respiratory epithelium by allergen-derived proteases, though it remains unclear whether gastric enzymes such as pepsin or trypsin exert similar effects on the esophageal epithelium [31].

The divergent actions of TSLP are linked to two isoforms: a short form with homeostatic functions and a long form activated during inflammation [32]. TSLP activity appears to be tissue-specific—pro-inflammatory in the skin and lungs, but regulatory in the gut and thymus. Reduced TSLP levels have been associated with Th1-driven diseases (e.g., Crohn’s disease), whereas elevated levels are characteristic of Th2-driven conditions (e.g., ulcerative colitis) [32].

Studies in eosinophilic esophagitis (EoE) have shown increased TSLP expression in esophageal tissue with basophil and mast cell infiltration [33], though other findings did not confirm this [34]. In GERD, our results demonstrate significantly higher TSLP levels in children, independent of reflux type, suggesting that TSLP release may result from epithelial injury caused by gastric contents regardless of acidity.

If confirmed, the role of TSLP in GERD could support its use as a diagnostic biomarker and as a therapeutic target, as has been proposed in asthma [35,36,37]. In our study, no associations were observed between TSLP and bronchial hyperresponsiveness or allergen sensitization, which may reflect the limited sample size [38].

### 4.2. IL-25 and IL-33

In contrast to TSLP, no statistically significant differences in IL-25 concentrations were observed between the two groups. However, the presence of IL-25 was associated with the type of reflux: higher IL-25 levels were noted in children with non-acidic GER compared to those with acidic GER. This may indicate that while IL-25 may not be involved in the pathogenesis of GERD, the components of non-acid reflux may have some role in its induction. In addition, IL-25 levels were not related to BHR or sensitization in the children studied.

Previous studies indicate that IL-25 has a proven role in asthma, and research is currently underway to assess its importance in other conditions, such as atopic dermatitis (AD) and inflammatory bowel disease (IBD) [39]. Like TSLP, IL-25 is also thought to be involved in maintaining intestinal homeostasis: IL-25 levels are significantly reduced both in the serum and the intestinal mucosa of patients with active IBD [34]. Studies suggest that, like TSLP, IL-25 plays a dual role in regulating the immune response during the development of autoimmune diseases [40].

No relationship was found between GERD and IL-33. Again, this may be due to the small number of subjects in the study, or its lack of involvement in the pathogenesis of GERD.

### 4.3. Periostin

Similarly, as periostin is known to play a role in the development of asthma, one of the aims of the study was to identify any potential relationship between periostin levels and the presence of GERD in children. Although no such association was observed, a significant correlation was found between periostin levels in children with GERD and the co-occurrence of sensitization to inhalant allergens: children with GERD and coexisting sensitization had higher serum periostin levels than those with GERD but without sensitization. In addition, higher periostin levels were noted in sensitized children with GERD, although periostin level was not found to be associated with the degree of sensitization. The explanation may lie in the accumulation of two trigger factors: the possible epithelial damage caused by GERD, and the potential influence of GERD on the development of sensitization to inhalant allergens.

In our study, the significant increase in periostin levels in children with GERD who were sensitized to inhalant allergens (asIgE positive) may reflect the biological role of periostin as a downstream mediator of type 2 inflammation. Periostin is an extracellular matrix protein induced by IL-4 and IL-13, and its expression is closely linked with airway remodeling, eosinophilic inflammation, and chronic allergic responses, as demonstrated by Takayama et al. [41] and further reviewed by Sonnenberg-Riethmacher et al. [42]. One possible mechanism is that GERD-related epithelial injury facilitates enhanced allergen penetration and Th2 immune activation, thereby amplifying periostin production; this concept was supported by van Rhijn et al. [43]. In addition, periostin itself may contribute to a feed-forward loop by promoting eosinophil recruitment and fibroblast activation, as shown by Masuoka et al. and by Zhernov et al., who reported that periostin enhances eosinophil adhesion and induces keratinocyte production of TSLP [44,45]. This synergy between epithelial damage and allergen-driven immune responses may partly explain why periostin levels were higher in sensitized children with GERD, suggesting its potential role as a biomarker linking reflux-related epithelial stress with allergic sensitization.

While our present data indicate that obesity was almost three times more common in children with GERD, as noted previously [15,16], no relationship was observed between obesity and periostin level, unlike Matsumoto et al. [46] and Kimura et al. [17].

Interestingly, in our cohort, periostin levels decreased in children sensitized to more than five allergens, compared with those sensitized to fewer allergens. This finding is contrary to the expectation that multiple sensitizations would be associated with progressively higher periostin concentrations as a marker of type 2 inflammation. Several explanations may be considered. First, this result may reflect a downregulation phenomenon, in which chronic and extensive allergen exposure induces compensatory mechanisms that limit periostin expression. Second, clinical heterogeneity (e.g., differences in the types of allergens, concomitant obesity) may have influenced periostin levels in this subgroup. Finally, the relatively small number of children with >5 sensitizations might have contributed to this unexpected result and increased variability. To our knowledge, similar observations have not been described in pediatric populations, and future studies with larger sample sizes are needed to clarify this intriguing association. In addition to alarmins, recent work (2021–2023) has highlighted a growing pipeline of non-invasive biomarkers for pediatric GERD—including salivary pepsin, exhaled breath metabolites (volatile sulfur compounds, acetic acid), serum inflammatory mediators (e.g., TNF-α), and oral microbiome signatures—although most candidates still lack pediatric validation and standardization. Current pediatric reviews continue to position pH impedance as the diagnostic reference while regarding biomarkers as adjunctive and investigational [27,47,48].

### 4.4. Strengths and Limitations

The present study has two key strengths, viz. that its analysis of cytokine levels (TSLP, IL-33, IL-25, periostin) was performed in children with GERD diagnosed by pH impedance, and that it includes an assessment of BHR and sensitization to inhalant allergens. To date, no such studies have been published. However, the study also has its limitations. Firstly, the study groups were modestly sized, especially the control group. Secondly, the control group was not selected on the basis of testing, due to the inability to perform invasive tests in healthy, asymptomatic children. Furthermore, although two isoforms of TSLP are known to exist, our study only determined the total TSLP concentration, without any detailed analysis of the isoforms. Finally, alarmin levels were only assessed in blood serum and not in biopsy specimens, which would have yielded more reliable data; however, such studies would require invasive tests, which are difficult to perform in children, especially healthy ones.

Given these limitation, future multicenter studies with larger cohorts are strongly recommended to validate our findings and provide more robust evidence. Beyond epithelial alarmins, several non-invasive biomarkers have recently been proposed in pediatric GERD. A 2023 systematic review synthesized multi-omic, multicompartmental candidates and reported moderate diagnostic performance for salivary pepsin, signal for exhaled breath volatiles and acetic acid, and elevated serum TNF-α—while emphasizing high risk of bias and heterogeneity across studies [47]. In children, salivary pepsin has correlated with symptoms and histologic esophagitis in esophageal-atresia cohorts, and peripheral hematologic indices may reflect disease activity in subsets [49]. Our finding of elevated TSLP therefore complements this emerging panel and supports future multi-marker, pediatric-focused validation studies; however, current guidelines and reviews still consider such biomarkers investigational and not yet ready for routine clinical decision-making [27,50].

## 5. Conclusions

Among the examined alarmins, only TSLP was shown to be potentially involved in the pathomechanism of GERD. TSLP levels were significantly higher in children with GERD than in those without GERD. These findings suggest that TSLP may represent a biomarker of epithelial immune activation in pediatric GERD. IL-25, IL-33 and periostin levels did not differ between groups. However, periostin levels were found to be associated with sensitization to inhalant allergens in children with GERD, as previously demonstrated in other studies; hence, periostin may play a role in the development of allergic sensitization. Nevertheless, further research is needed to clarify the pathomechanism of GERD and support the development of new treatment options.

## Figures and Tables

**Table 1:** Inclusion and exclusion criteria for the study group and control group.

Inclusion Criteria for the Study Group	Inclusion Criteria for the Control Group
age 7–17 years old	
suspicion of GERD based on presenting symptoms	
determination of gastroesophageal reflux based on the result of 24-h esophageal pH impedance monitoring	the absence of the gastroesophageal reflux (determined by the result of 24-h esophageal pH impedance monitoring)
**Exclusion Criteria for the Study and Control Group**	
age < 7 years old or >17 years old	
chronic diseases that may have a significant impact on the outcome of the study (like diabetes, allergic diseases)	
the presence of an infection in the last 6 weeks	
asthma	
the presence of contraindications to carrying out the planned procedures	
inability of the respondent to understand procedures and cooperate	

**Table 2:** Characteristics of the study group and the control group.

Parameters	Children with GERD *n* = 68 (73.1%)	Children Without GERD *n* = 25 (26.9%)	*p*
Age [years] (mean ± standard deviation)	10.4 ± 3.3	10.8 ± 4.0	0.605
Body weight [kg] (mean ± standard deviation)	38.6 ± 14.9	37.1 ± 13.5	0.433
BMI [kg/m^2^] (mean ± standard deviation)	18.7 ± 3.7	18.2 ± 3.5	0.321
Gender [*n*; %]:			
Boys	40; 58.8%	12; 48%	0.276
Girls	28; 41.2%	13; 52%	
Place of residence [*n*; %]:			
village	24; 35.3%	9; 36%	0.831
small city (<20 thousand residences)	15; 22.1%	5; 20%	
medium city (20–100 thousand residences)	9; 13.2%	4; 16%	
large city (>100 thousand residences)	20; 29.4%	7; 28%	
Having siblings—yes [*n*; %]	53; 77.9%	18; 72%	0.583

**Table 3:** TSLP, IL-25, IL-33, and periostin levels in children with and without GERD according to the ascendancy of bronchial hyperresponsiveness (BHR+/BHR−).

Tested Parameter	Children with GERD (*n* = 68)			Children Without GERD (*n* = 25)		
	BHR+ (*n* = 22; 32.4%)	BHR− (*n* = 46; 67.6%)	*p* Value	BHR+ (*n* = 7; 28.0%)	BHR− (*n* = 18; 72.0%)	*p* Value
TSLP [pg/mL]						
average ± standard deviation	20.32 ± 10.93	21.14 ± 12.89	0.478	17.34 ± 4.55	14.19 ± 8.71	0.426
min–max	3.10–50.12	3.10–74.08		10.94–22.53	0.88–33.96	
IL-25 [pg/mL]						
average ± standard deviation	16.53 ± 16.34	12.28 ± 13.28	0.262	8.52 ± 4.79	7.90 ± 8.71	0.199
min–max	1.252–72.46	1.08–58.99		1.43–16.71	1.25–34.61	
IL-33 [pg/mL]						
average ± standard deviation	7.01 ± 4.64	6.77 ± 5.04	0.468	6.84 ± 3.87	5.96 ± 4.46	0.532
min–max	1.31–17.77	0.70–20.27		0.59–11.17	0.12–16.05	
periostin [pg/mL]						
average ± standard deviation	6.21 ± 3.55	5.77 ± 2.19	0.531	5.93 ± 0.52	5.31 ± 1.60	0.325
min–max	1.74–41.00	0.74–14.81		4.91–6.42	1.49–8.15	

**Table 4:** TSLP, IL-25, IL-33 and periostin levels in relation to the presence of sensitization to airborne allergens (SPT) in children with and without GERD.

Tested Parameter	Children with GERD			Children Without GERD		
	Sensitized (*n* = 35; 51.5%)	Not Sensitized (*n* = 33; 49.5%)	*p* Value	Sensitized (*n* = 11; 44.0%)	Not Sensitized (*n* = 14; 56.0%)	*p* Value
TSLP [pg/mL]	18.21 ± 9.97	22.12 ± 10.61	0.125	13.17 ± 9.28	16.57 ± 8.19	0.340
IL-25 [pg/mL]	11.71 ± 14.15	15.56 ± 14.44	0.274	8.33 ± 6.30	7.87 ± 8.91	0.886
IL-33 [pg/mL]	5.88 ± 4.29	7.87 ± 5.32	0.093	6.85 ± 4.91	5.70 ± 3.75	0.511
periostin [pg/mL]	5.95 ± 2.92	5.86 ± 2.46	0.891	5.44 ± 1.77	5.52 ± 1.77	0.898

**Table 5:** TSLP, IL-25, IL-33 and periostin levels in relation to the presence of sensitization to airborne allergens (asIgE) in children with and without GERD.

Tested Parameter	Children with GERD			Children Without GERD		
	Sensitized (*n* = 17; 25.0%)	Not Sensitized (*n* = 51; 75.0%)	*p* Value	Sensitized (*n* = 10; 40.0%)	Not Sensitized (*n* = 15; 60.0%)	*p* Value
TSLP [pg/mL]	20.98 ± 8.80	19.78 ± 10.95	0.684	17.74 ± 10.26	13.30 ± 7.26	0.217
IL-25 [pg/mL]	17.20 ± 18.20	12.49 ± 12.88	0.253	9.19 ± 5.42	7.32 ± 9.04	0.563
IL-33 [pg/mL]	7.41 ± 4.30	6.67 ± 5.09	0.591	7.79 ±4.49	5.14 ± 3.89	0.129
periostin [pg/mL]	7.61 ± 4.11	5.34 ± 1.72	0.002	5.75 ± 0.86	5.30 ± 1.67	0.442

**Table 6:** Average concentrations: TSLP, IL-25, IL-33 and periostin in relation to the amount of airborne allergen sensitization (SPT, asIgE) present in the children studied.

Tested Parameter (Average ± SD)	SPT				asIgE			
	Sensitization to 1 Allergen	Sensitization to 2–5 Allergens	Sensitization to >5 Allergens	*p* Value	Sensitization to 1 Allergen	Sensitization to 2–5 Allergens	Sensitization to >5 Allergens	*p* Value
TSLP [pg/mL]	14.0 ± 7.8	17.8 ± 10.3	16.4 ± 11.8	0.231	25.1 ± 7.7	19.8 ± 9.1	10.3 ± 5.4	0.244
IL-25 [pg/mL]	5.5 ± 5.3	18.9 ± 37.7	7.4 ± 3.0	0.456	13.8 ± 8.2	28.4 ± 52.6	7.3 ± 3.8	0.085
IL-33 [pg/mL]	4.8 ± 5.2	6.4 ± 4.4	6.6 ± 4.7	0.563	8.6 ± 4.9	7.3 ± 4.3	6.9 ± 3.8	0.626
periostin [pg/mL]	5.1 ± 1.3	6.1 ± 2.9	5.3 ± 1.8	0.709	6.8 ± 1.3	7.6 ± 4.1	4.5 ± 1.5	0.003

## Data Availability

The data can be made available upon reasonable request—please contact the correspondence author. The data are not publicly available due to the fact that they contain information that could compromise the privacy of research participants.

## Abbreviations

The following abbreviations are used in this manuscript:

AD	atopic dermatitis
asIgE	allergen-specific IgE
ATS	American Thoracic Society
BHR	bronchial hyperresponsiveness
CD	Crohn’s Disease
EAACI	European Academy of Allergy and Clinical Immunology
EoE	eosinophilic esophagitis
GER	gastro-esophageal reflux
GERD	gastro-esophageal reflux disease
GerdQ	Gastroesophageal Reflux Disease Questionnaire-Q
IBD	inflammatory bowel disease
IL-25	interleukin 25
IL-33	interleukin 33
ISAAC	International Study of Asthma and Allergies in Childhood
SAP	Symptom Association Probability
SI	Symptom Index
SPT	skin prick test
SSI	Symptom Sensitivity Index
TSLP	thymic stromal lymphopoietin
UC	ulcerative colitis
